# Comparative Evaluation of Bone–Implant Contact in Various Surface-Treated Dental Implants Using High-Resolution Micro-CT in Rabbits’ Bone

**DOI:** 10.3390/ma17225396

**Published:** 2024-11-05

**Authors:** Rafał Zieliński, Adam K. Puszkarz, Tomasz Piętka, Jerzy Sowiński, Monika Sadowska-Sowińska, Agata Kołkowska, Wojciech Simka

**Affiliations:** 1 Stomatologia na Ksieżym Młynie, Lodz, 16D Tymienieckiego, 90-365 Lodz, Poland; 2 Textile Institute, Faculty of Material Technologies and Textile Design, Lodz University of Technology, 116 Żeromskiego Street, 90-924 Lodz, Poland; adam.puszkarz@p.lodz.pl; 3 Amigo Dental, Rojna St. 22, 91-128 Lodz, Poland; pietkatomasz@gmail.com; 4 Private Dental Clinic, Tetmajera 3A Rd, 05-080 Izabelin C, Poland; jersow@gmail.com (J.S.); msadowska.gdansk@gmail.com (M.S.-S.); 5 Department of Inorganic Chemistry, Analytical Chemistry, and Electrochemistry, Faculty of Chemistry, Silesian University of Technology, Krzywoustego St. 6, 44-100 Gliwice, Poland; agatkol653@student.polsl.pl; 6 Chemistry Students Research Society, Faculty of Chemistry, Silesian University of Technology, Strzody 9 St., 44-100 Gliwice, Poland

**Keywords:** osseointegration, X-ray microtomography, dental implant surface

## Abstract

This study evaluated the bone-to-implant contact (BIC) of various surface-treated dental implants using high-resolution micro-CT in rabbit bone, focusing on the effects of different treatments on osseointegration and implant stability before and after bone demineralization. Six male New Zealand White rabbits were used. Four implant types were tested: machined surface with anodizing, only etching, sandblasting with Al_2_O_3_ + etching, and sandblasting with TiO_2_ + etching. Implants were scanned with high-resolution micro-CT before and after demineralization. Parameters like implant volume, surface area, and BIC were determined using specific software tools. During demineralization, the BIC changed about 6% for machined surface with anodizing, 5% for only etching, 4% for sandblasting with Al_2_O_3_ + etching, and 10% for sandblasting with TiO_2_ + etching. Demineralization reduced BIC percentages, notably in the machined surface with anodizing and sandblasting with TiO_2_ + etching groups. Etching and sandblasting combined with etching showed higher initial BIC compared to anodizing alone. Demineralization negatively impacted the BIC across all treatments. This study underscores the importance of surface modification in implant integration, especially in compromised bone. Further research with larger sample sizes and advanced techniques is recommended.

## 1. Introduction

In both dentistry and orthopedics, evaluating the osseointegration of an implant is crucial for determining the stability of internal fixation. The bone-to-implant contact (BIC) ratio, which is the proportion of the length of bone in direct contact with the implant thread to the total length of the implant thread, is used to assess the level of osseointegration and the implant’s stability [1,2,3].

Currently, histomorphometry is regarded as the gold standard for analyzing BIC [4]. However, the process of creating histological slides is labor-intensive, time-consuming, and destructive. This method yields only a limited number of representative cross-sections from specific positions on the implant, which is insufficient for providing comprehensive information about the entire implant [4,5]. Furthermore, once a specimen has undergone histomorphometric analysis, it cannot be used for any additional assessments.

Micro-computed tomography (μCT) has been widely adopted for evaluating the structure of bony tissue due to its time-saving, convenient, and nondestructive nature. The μCT data can be reconstructed to examine bone architecture at any location around the implant and analyze bone parameters in the region of interest (ROI). However, the different attenuation coefficients of bone and implant materials generate metallic artifacts at the bone–implant interface, affecting the BIC measurements obtained using μCT [6,7,8]. Some studies recommend using soft filters, such as aluminum or brass, to reduce artifacts during CT scanning and employing correction functions in analysis software to mitigate these artifacts [9,10]. These functions include misalignment compensation, ring artifact reduction, and beam-hardening correction [11]. Additionally, a few studies suggest excluding several voxels near the screw surface to eliminate the artifact zone when measuring BIC [12,13]. Despite these efforts, there is currently no ideal method or program for accurately measuring BIC using μCT [14].

So far, bone-to-implant contact measurements have been derived from two-dimensional (2D) μCT images or three-dimensional (3D) μCT models using various methods. Researchers have explored the correlation between BICs obtained from μCT and histology to determine if μCT could replace histology in assessing osseointegration [10,15,16]. However, the results of these correlation studies have been inconsistent. Few studies have evaluated implant osseointegration by integrating 2D μCT slices from multiple locations, which could potentially offer a more comprehensive assessment [5].

The successful integration of dental implants into bone is characterized by three key criteria [17]. The absence of rejection reactions includes a lack of inflammatory responses in surrounding tissues, local necrotic changes, and systemic manifestations such as allergic or immune reactions. The formation of morphofunctional determinants in the contact area, known as the “implant–tissue medium”, involves bone or bone-like substances in the case of osseointegration. The relative stability of determinants, including mechanical stability, of these morphofunctional determinants over time reflects the dynamic equilibrium within the “implant–tissue substrate” system.

Therefore, the primary operational properties of dental implants, such as osseointegration and biocompatibility, largely depend on the characteristics of their surface layer, which interacts directly with the biological tissues of the body (bone and gingiva). Consequently, surface modification of dental implants is crucial, and many scientific studies focus on this aspect. Surface modification methods can be broadly categorized into groups based on their primary effect, mechanical, physical, chemical, or biochemical, and combined methods that utilize multiple types of processing exist.

Mechanical treatment is one method of surface treatment which includes, among others, machining through cutting (machine stroking) [18,19,20,21,22,23] and sandblasting. The main goal of these treatments is to enhance the bone’s adherence to the implant (osseointegration), which is crucial for the long-term success of implantation.

Sandblasting is simple and cost-effective, enhancing cell adhesion and proliferation and osteoblast differentiation. However, acid etching of the implant is often required to homogenize the surface micro-profile and remove any remaining sand particles. If not performed, the surface material’s inhomogeneity can reduce the implant’s corrosion resistance [24].

Physical treatment techniques involve laser ablation [25,26], plasma spraying [27], vacuum arc coating [28,29,30], glow discharge plasma [31], ultraviolet light treatment [32], and ion implantation [33]. Chemical surface treatment methods are techniques that modify the properties of material surfaces using chemical reactions. Their goal is to improve surface durability, corrosion resistance, adhesion, aesthetics, or biocompatibility. These include anodizing, chemical etching, sandblasting, electrochemical [34,35] and electrophoretic [36] deposition of coatings, the sol–gel method [37], and plasma electrolytic oxidation [38].

Anodization is commonly used in bioengineering to modify surfaces and improve the properties of implants by forming an oxide layer. The thickness of this layer follows Faraday’s law. Titanium alloys are anodized to thicken the natural TiO_2_ oxide layer. Anodizing increases the surface roughness of titanium, creating an oxide layer with a crystalline structure primarily composed of anatase TiO_2_. The increased wettability and microporous structure enhance the adhesion of specific proteins, improving osseointegration and surface bioactivity. The process is conducted in oxidizing acid solutions (sulfuric acid, phosphoric acid) or alkaline phosphate or silicate solutions, with varying concentrations and process conditions (voltage, current density) [39,40].

Chemical (including electrochemical) etching can be divided into two main groups depending on the solution used: acid etching and alkaline etching (alkali treatment). Different surface textures and roughness can be achieved by varying the composition of etching solutions (typically the acid concentration), the temperature, and etching duration. Acid etching notably increases the roughness of the implant surface without altering its contact angle. In contrast, alkaline etching enhances the hydrophilicity of the dental implant surface. Additionally, acid etching is performed at lower temperatures and requires less time compared to the standard alkaline etching treatment [41].

Sandblasting of the surface followed by acid etching combines the benefits of both surface modification techniques. The SLA (Sand-blasted, Large-grit, Acid-etched) surface features a coarse-grained texture, resulting in improved osseointegration and osseoconductivity, particularly in the early stages after implantation, compared to untreated surfaces. The RBM (Resorbable Blast Media) surface is created by sandblasting the implant with resorbable Ca–phosphate compound particles, followed by etching in a low-concentration organic acid. This process produces deeper micropores compared to SLA, enhancing osseoconductivity and helping to reduce osteoporosis. However, the combined treatment results in a hydrophobic surface, which can slightly hinder the osseointegration of implants [42,43].

The aim of this article is to investigate the effects of different surface treatments on the osseointegration and stability of dental implants by comparing BIC values before and after bone demineralization using high-resolution µCT analysis and to examine whether selected innovative surface treatments, including etching and sandblasting, influence the degree of demineralization in cells surrounding implants. An innovative aspect of this publication is the use of an alternative type of surface treatment, coupled with in vivo studies that have been conducted to assess its effects. The following research null hypotheses were raised: (a) the method of preparing the implant surface does not affect BIC; (b) µCT is not a tool for correct assessment of BIC.

## 2. Materials and Methods

### 2.1. Implant Preparation

The implants were made of grade 4 titanium using CNC machines. They were then washed according to the company’s procedures, and surface treatment began—sandblasting and etching. Implants were etched in 50% H_2_SO_4_. Sandblasting was used in addition for some of them. Two types of SLA (Sandblasted, Large grit, Acid-etched) surfaces were obtained. First, part of implants were sandblasted with Al_2_O_3_ (grain size 180–250 µm) and etched in 50% H_2_SO_4_ at 120 °C. The rest of the samples were sandblasted with TiO_2_ (grain size 106–150 µm) and etched in 50% H_2_SO_4_ at 120 °C. After surface treatment, the samples were rinsed in distilled water and air-dried. The choice of compounds used for sandblasting was deliberate. Studies confirm the positive influence of sandblasting with TiO_2_ [44,45,46] and Al_2_O_3_ [47] on bone–implant contact (BIC) as early as the beginning of the 21st century. Additionally, Al_2_O_3_ is the most commonly used abrasive in sandblasting processes, while TiO_2_ is considered a more biocompatible alternative. These assumptions were verified through a series of tests and analyses. The concentrations of acids applied for etching were selected experimentally in preliminary studies.

### 2.2. In Vivo Tests

Our research was conducted on 6 male New Zealand White rabbits, each under 12 weeks of age and weighing no less than 3.5 kg. This study was approved by the Local Ethics Committee under the number 18/ŁB195/2021. All animals were quarantined before the start of the research and subjected to a one-day handling acclimation period.

The European rabbit animal model was chosen in accordance with the requirements and standards of PN-EN ISO 10993-6:2017-04, Biological Evaluation of Medical Devices—Part 6: Tests for Local Effects after Implantation. Dental implants were deliberately designed and manufactured for rabbits purposes. The dimension of the implants was 2.5 mm × 6 mm. The first and final drill was 1.6 mm in diameter.

In the initial stage, we established the procedure techniques, implantation sites, and drills using a dead rabbit. Only then did we proceed to in vivo studies. There were some changes in the number of rabbits used, but for economic and ethical reasons, we aimed to limit their number to 6. Additionally, we used 2 rabbits designated for practicing cutting bone into pieces and taking RVG (radiovisiography) and CBCT (cone beam computed tomography) images to minimize the risk of damaging the valuable material with implants.

### 2.3. Scanning Electron Microscopy

The surface of all samples was thoroughly analyzed for morphology using a scanning electron microscope (SEM) both before and after demineralization (Phenom ProX; ThermoFisher Scientific, Waltham, MA, USA). Surface morphology analysis is achieved by scanning the sample with a focused beam of electrons, which are emitted by the cathode and shaped into a beam by the optical system. The emitted electron signal is processed to generate an SEM image. The topographic contrast in the image is produced by the emission of secondary electrons. For this analysis, an accelerating voltage of 15 kV was applied.

### 2.4. Micro-Computed Tomography

**Figure 1:**
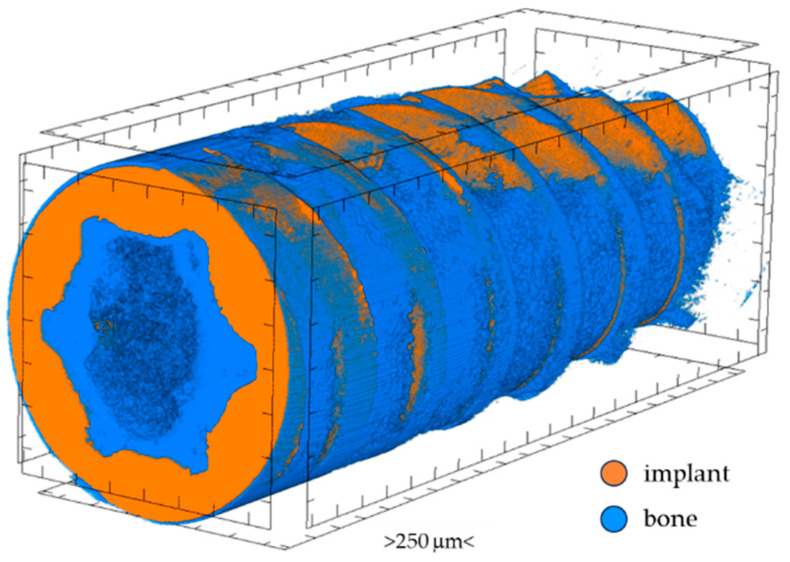
Three-dimensional visualization of implant with bone attached.

High-resolution X-ray computed tomography, µ-CT (SkyScan 1272; Bruker, Kontich, Belgium), was used to investigate the geometric parameters (implant volume, implant surface, BIC) of the four tested implants. Implants screwed into the bone were scanned twice (before and after bone demineralization) under identical conditions: X-ray source voltage: 50 kV; X-ray source current: 200 µA; pixel size: 5 µm; rotation: 180° rotation; rotation step: 0.2°, with an Al 0.5 + Cu 0.038 filter. Using the software NRecon 1.7.4.2, CTAn 1.17.7.2+, Data Viewer 1.5.6.2 (Bruker, Kontich, Belgium), and ImageJ (ver. 1.8.0_245), the following implant parameters were determined: (1) implant volume, (2) implant surface, and (3) BIC. Using CTvox 3.3.0 r1403 software (Bruker, Kontich, Belgium), three-dimensional visualizations of each implant with bone attached were made (Figure 1).

**Figure 2:**
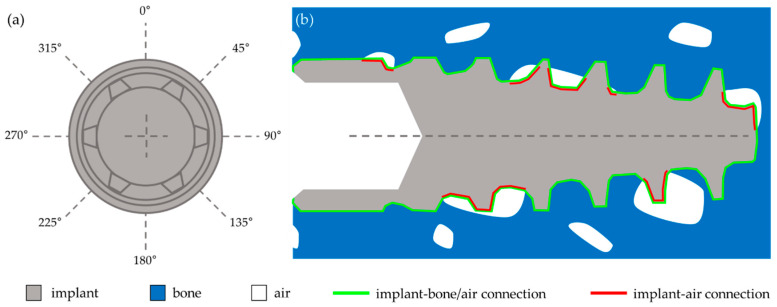
A schematic illustration of the BIC calculation method.

Figure 2 shows the method based on which *BIC* was determined. Figure 2b shows a cross-section in the 0°–180° plane of the implant screwed into the bone (Figure 2a). The green line (length *L*_green_) marks the edge of the implant in this selected plane, including the implant–bone connection and the implant–air connection (in the place where the bone has not attached to the implant). The red line (length *L*_red_) marks the line covering the implant–air connection. In this case, *BIC* was calculated using Formula (1).

(1) $$BIC=\frac{L_{green}-L_{red}}{L_{green}}\times 100\%$$

The average BIC value for each implant was calculated from four BIC values calculated for sections in four planes (0°–180°; 45°–225°; 90°–270°; 135°–315°). The orientation of each of the four implants in any plane was randomly selected to better average the obtained BIC results.

**Figure 3:**
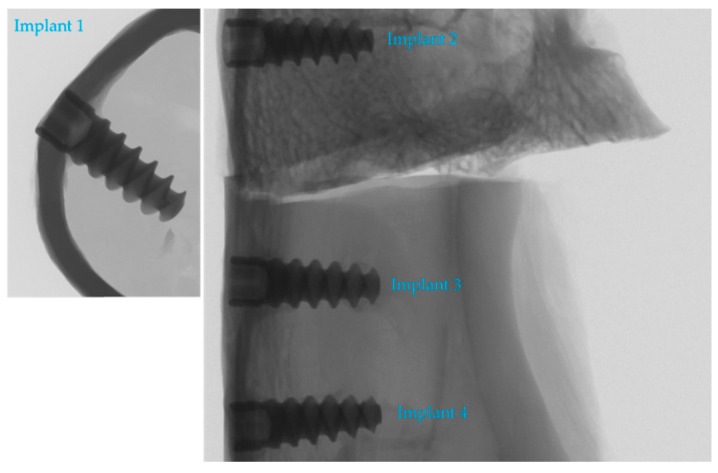
Four tested implants screwed into rabbit’s bone. Implant 1—machined surface with only anodizing; Implant 2—only etching; Implant 3—sandblasting by Al_2_O_3_ + etching; Implant 4—sandblasting by TiO_2_ + etching.

Figure 3 shows the RVG (Gendex Dental Systems, Hatfield, PA, USA) of all four dental implants placed in a rabbit’s tibia and femoral bone.

Figure 3 shows that the implants were properly screwed into the bone. There are no visible changes around them that would indicate a problem with osseointegration.

## 3. Results

**Figure 4:**
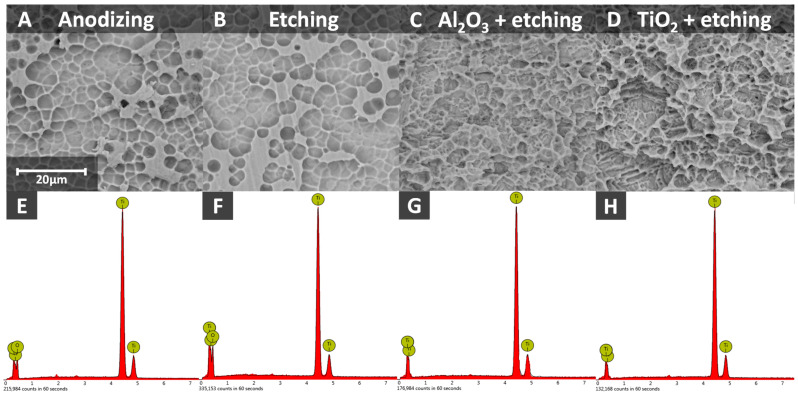
SEM (**A**–**D**) images and EDX spectra (**E**–**H**) of samples after different surface treatment processes.

**Figure 5:**
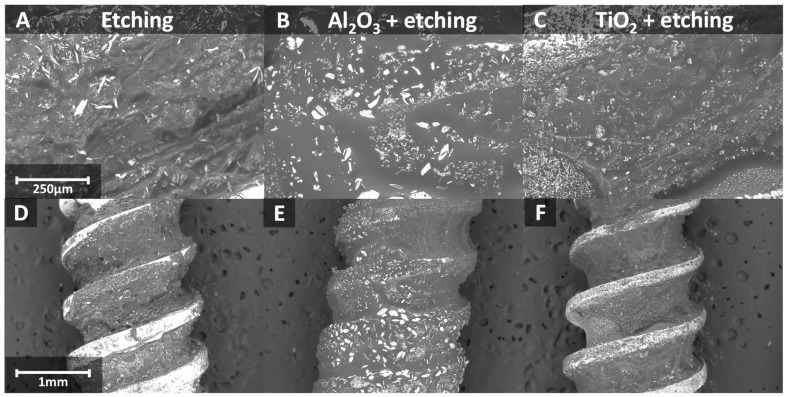
SEM images of implants after demineralization (**A**–**C**—magnification ×200; **D**–**F**—magnification ×50).

The samples after surface treatment were thoroughly examined. An analysis was conducted using a scanning electron microscope (SEM) with energy-dispersive X-ray spectroscopy (EDX) before demineralization to compare the surface properties and morphology of implants treated in different ways (Figure 4). After demineralization, another series of SEM images was taken to assess the changes resulting from demineralization (Figure 5). This allows for the observation of differences in surface characteristics, while EDX spectra identify all elements present on the sample surfaces. The information provided by this analysis is highly valuable for selecting the appropriate surface treatment method.

Energy-dispersive X-ray spectroscopy undoubtedly revealed titanium as the main element (Figure 4). Etching in 50% H_2_SO_4_ did not cause any noticeable changes in morphology, as observed in the SEM images. However, the EDX spectra show a higher oxygen peak. Sandblasting and etching produced a similar effect. The surface is covered with small pits with sharp edges, which are deeper in the sample sandblasted with titanium oxide. In the sandblasted and etched samples, no oxygen was detected. This study provides preliminary insights into expected outcomes following the implantation of similarly treated implants, considering surface morphology and characteristics.

After implant insertion, significant demineralization of the implants was observed on SEM images (Figure 5).

A significant amount of tissue growth was observed around each of the previously modified implants. The osseointegration process is advanced and clearly visible in all samples. The best result is achieved with the implant that was sandblasted with Al_2_O_3_ and then etched, where the tissue fully covers the implant (Figure 5). Observing implants post-implantation allows us to evaluate the effect of osseointegration, enabling a comparison of the efficacy of osseointegration across implants subjected to various surface treatments.

Table 1, Table 2, Table 3 and Table 4 present the results of implant connections with the edge, air, and bone. For each implant, the average bone-to-implant contact (BIC) is also provided. This research represents a highly detailed analysis of the quality of the bond between the implant and the bone.

Based on the micro-CT results obtained from dental implants placed in rabbits, several observations and implications can be drawn regarding the bone-to-implant contact (BIC) percentages before and after demineralization across different surface treatments. Given the ethical constraints limiting the sample size to six rabbits, this discussion focuses on the trends and patterns observed while acknowledging the limitations in statistical power.

The results summarized in Table 1, Table 2, Table 3 and Table 4, obtained from the measurements after demineralization, exhibit a greater amplitude compared to those measured prior to demineralization. The standard deviation of the post-demineralization samples is approximately twice as large as that of the pre-demineralization samples. The greatest difference in the results was observed for the implants that underwent sandblasting with Al_2_O_3_ and acid etching. The lower standard deviations before demineralization indicate more consistent outcomes following the initial treatments. In contrast, the relatively higher standard deviations after demineralization, particularly in the sandblasting groups, suggest increased variability in response to demineralization. This variability may be attributed to differences in individual bone quality and healing responses among the rabbits. The unique characteristics of each organism could have contributed to a high standard deviation. The variation in BIC values across different measurement points may also result from surface heterogeneity. Surface treatments—etching or sandblasting—may not have produced identical effects across all areas. These inconsistencies could have been challenging or impossible to detect during the analysis of other research findings.

Moreover, the increased variability post-demineralization underscores the need for further research to better understand the underlying mechanisms and to develop strategies to mitigate these effects.

The BIC analysis enabled a quantitative assessment of osseointegration quality, highlighting the variability in the results. This study identified limitations arising from the sample size and variations in the bone characteristics of each rabbit. This is the most precise technique among those used in the current study to determine the degree of osseointegration.

**Figure 6:**
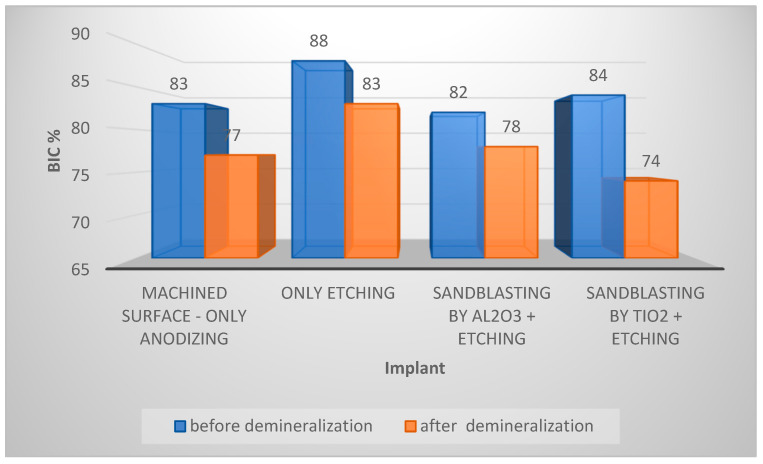
Average BIC value for all implants tested.

Figure 6 presents the average bone-to-implant contact (BIC) values for implants subjected to various surface treatments, both before and after demineralization. This comparison makes differences in BIC more readily observable.

The highest BIC values are observed in Figure 6 for the implant that was only etched. The BIC value for each implant decreased by 4–10% after demineralization. This indicates that demineralization adversely affects the bone–implant interface, potentially compromising implant stability. The largest BIC reduction was seen in the implant sandblasted with TiO_2_ and etched, while the smallest reduction occurred in the implant sandblasted with Al_2_O_3_ and etched. Titanium oxide (TiO_2_) is softer than aluminum oxide (Al_2_O_3_), which may have resulted in less effective sandblasting. The difference in grain hardness could have influenced surface roughness, leading to uneven etching. Overall, sandblasting reduced BIC compared to the etched-only implant. Despite the reduction in BIC during demineralization, the etched-only implant maintained a BIC level similar to that of the other implants before demineralization.

The analysis of surface area and volume for all implants (Table 5) allows us to assess mass loss or gain, as well as changes in surface area. Such changes in implant characteristics can lead to varying outcomes, making it crucial to monitor these parameters. Significant differences in mass may prevent the proper fit of different components, potentially leading to loosening. Conversely, changes in surface area impact the quality of the implant–bone connection, where a larger surface area is preferred.

The volumes of the implants across the different treatments are relatively consistent, ranging from 8.91 mm^3^ to 9.54 mm^3^ (Table 5). Implant volume appears to be largely unaffected by the surface treatment method. The slight variations in volume suggest that the chosen treatments do not significantly alter the overall dimensions of the implants.

The surface areas of the implants vary more significantly between treatments. The etching-only method resulted in the highest surface area (69.11 mm^2^), while the anodizing-only method resulted in the lowest (60.82 mm^2^). Sandblasting followed by etching with either Al_2_O_3_ or TiO_2_ produced intermediate surface areas (66.85 mm^2^ and 63.29 mm^2^, respectively). This suggests that the combination of sandblasting and etching effectively increases the surface area compared to anodizing alone. The increased surface area observed in the etched and sandblasted samples may contribute to better osseointegration, as a larger surface area typically enhances the mechanical interlocking between the implant and bone.

## 4. Discussion

Research indicates that the best osseointegration results are achieved with sandblasting and etching, as these treatments result in minimal mass loss while effectively increasing surface area, thereby enhancing the implant’s integration with bone. Surface treatment is a critical process that often determines the quality of the bone–implant interface. Selecting the appropriate type of surface treatment and optimizing the parameters of the procedure have a significant impact on bone-to-implant contact (BIC) values and the potential risk of implant failure. Studies have demonstrated that the rough surface resulting from etching and sandblasting promotes bone tissue integration with the implant. Selecting compounds for this type of treatment is an essential first step. According to the literature, sandblasting with aluminum oxide (Al_2_O_3_) is the most common method. However, TiO_2_ may be a beneficial alternative due to its positive effects on osteoblast-like cells and bone integration. The SLA method with Al_2_O_3_ and sulfuric (H_2_SO_4_) or hydrochloric (HCl) acid is well documented in the literature, although there are mentions of potential risks for long-term osseointegration due to particle embedding on the surface during treatment. Unfortunately, there is a lack of in vivo studies documenting the results of using TiO_2_ in SLA surface preparation [48,49,50].

The results found in the literature regarding Al_2_O_3_ use are inconsistent. Some studies suggest it reduces osteolysis without a significant impact on osteoblast-like or macrophage-like cells. Other studies indicate that Al_2_O_3_ induces an inflammatory response during the co-culture of osteoblasts and macrophages in its presence, potentially due to the release of cytokines, which act as inflammatory mediators. Sandblasting with Al_2_O_3_ may cause its accumulation at the mineralization front and within the osteoid matrix itself [51,52,53,54,55]. Despite these drawbacks, Al_2_O_3_ remains one of the only ceramic oxides that produces satisfactory results, leading researchers to seek better alternatives. TiO_2_ minimizes the risk of contamination by residual debris from the blasting procedure. While many studies present biomechanical results, few address in vivo studies. It has been confirmed that TiO_2_ sandblasting increases wettability compared to a machined surface, positively influencing the osseointegration process. Accelerated bone formation and improved interface quality have also been confirmed [49,56,57].

One significant association with implant insertion is the process of demineralization caused by inflammation, where hydroxyapatite (HA) mineral ions are removed from hard tissues, especially bone. Demineralization can be triggered by various factors, including uneven bone loading due to the implant. Furthermore, even minimal implant movement can damage bone, leading to implant loosening. Upon implant placement, there is an increased risk of bacterial inflammation, which may occur more frequently. Bone demineralization alters the bone’s chemical composition and physicochemical properties. The production of osteoblasts may also be impaired, resulting in lower BIC values post-demineralization (as observed in implants treated with TiO_2_ sandblasting). Deterioration in bone quality around the implant increases the risk of complications due to unstable implant integration with bone [58,59,60].

The conducted studies provide insights into the surface characteristics and morphology of BIC, as well as in vivo results from studies on rabbits. A detailed analysis confirmed the positive effects of surface treatment on successful implantation. An irregular surface improved the bone–implant interface quality. The BIC values achieved for etching and TiO_2_ sandblasting were higher than those for Al_2_O_3_ before demineralization. After demineralization, the opposite effect was observed, indicating that TiO_2_ is a suitable alternative to Al_2_O_3_ as a sandblasting abrasive. The results show that demineralization reduced BIC, with the most significant impact seen in the TiO_2_ sandblasted implants.

This study aimed to highlight differences in bone-to-implant quality using various surface treatment methods. The in vitro and in vivo studies were conducted on rabbit implants and cannot be directly applied to human implants, which are larger. A series of experiments is necessary to optimize the parameters and control the applied methods for human application.

The limited sample number (n = 6) and size restricts the ability to generalize the findings. Larger studies are necessary to validate these results and establish more robust statistical conclusions. Future research should also focus on the long-term outcomes of these surface treatments to determine their durability and effectiveness over time, particularly in the face of challenges like demineralization. Employing advanced analytical techniques and histological evaluations could provide deeper insights into bone–implant interactions and the mechanisms driving the observed changes in BIC percentages.

The current study provides valuable preliminary insights into the effectiveness of different surface treatments on dental implants; however, the ethical and methodological limitations necessitate cautious interpretation of the results. Continued research with larger sample sizes and comprehensive analyses is essential to optimize implant surface technologies for improved clinical outcomes.

## 5. Conclusions

The high-resolution micro-CT analysis provided detailed insights into the effects of different surface treatments on dental implant integration. The current study found that while all surface treatments facilitated strong initial bone–implant contact, demineralization significantly impacted the stability and variability of this contact. The data suggest that surface treatments such as etching and sandblasting combined with etching generally lead to higher initial BIC percentages compared to anodizing alone. Etching alone shows the highest BIC percentage before demineralization, indicating its effectiveness in promoting bone integration. The SEM images showed that all of the implants were demineralized, with the best result obtained for the sample etched and sandblasted with Al_2_O_3_. Considering the results of the in vivo studies, SEM image analysis, and bone-to-implant connection metrics, etching and sandblasting with Al_2_O_3_ represent the most effective combined surface treatment method.

These findings highlight the importance of surface treatment in enhancing implant stability, particularly in conditions where bone quality is compromised. Further research is recommended to develop and optimize surface treatments that improve long-term implant integration in demineralized bone conditions.

## Figures and Tables

**Table 1:** Average BIC value for implant with machined surface.

	Machined Surface—Only Anodizing							
	Before Demineralization				After Demineralization			
cross-sectional plane	0°–180°	45°–225°	90°–270°	135°–315°	0°–180°	45°–225°	90°–270°	135°–315°
implant–edge connection line [mm]	16,784	16,606	16,523	15,535	16,151	16,934	16,812	15,303
implant–air connection line [mm]	2432	2853	4118	1748	2037	1768	6674	4702
implant–bone connection line (BIC) [%]	86	83	75	89	87	90	60	69
average BIC [%]	83				77			
standard deviation BIC [%]	6				14			

**Table 2:** Average BIC value for implant with etched surface.

	Only Etching							
	Before Demineralization				After Demineralization			
cross-sectional plane	0°–180°	45°–225°	90°–270°	135°–315°	0°–180°	45°–225°	90°–270°	135°–315°
implant–edge connection line [mm]	16,781	16,957	16,853	15,787	19,950	15,329	17,596	17,621
implant–air connection line [mm]	1942	2335	2379	1320	6741	1204	1458	2852
implant–bone connection line (BIC) [%]	88	86	86	92	66	92	92	84
average BIC [%]	88				83			
standard deviation BIC [%]	3				12			

**Table 3:** Average BIC value for implant with surface sandblasted by Al_2_O_3_ and etched.

	Sandblasting by Al_2_O_3_ + Etching							
	Before Demineralization				After Demineralization			
cross-sectional plane	0°–180°	45°–225°	90°–270°	135°–315°	0°–180°	45°–225°	90°–270°	135°–315°
implant–edge connection line [mm]	16,953	17,638	15,692	16,957	16,321	16,275	17,258	17,028
implant–air connection line [mm]	2976	1497	1299	6193	1490	1946	9137	2481
implant–bone connection line (BIC) [%]	82	92	92	63	91	88	47	85
average BIC [%]	82				78			
standard deviation BIC [%]	13				21			

**Table 4:** Average BIC value for implant with surface sandblasted by TiO_2_ and etched.

	Sandblasting by TiO_2_ + Etching							
	Before Demineralization				After Demineralization			
cross-sectional plane	0°–180°	45°–225°	90°–270°	135°–315°	0°–180°	45°–225°	90°–270°	135°–315°
implant–edge connection line [mm]	16,112	17,071	16,726	16,999	17,808	15,978	18,563	17,415
implant–air connection line [mm]	3051	1755	1848	3949	2008	4451	8646	2878
implant–bone connection line (BIC) [%]	81	90	89	77	89	72	53	83
average BIC [%]	84				74			
standard deviation BIC [%]	6				16			

**Table 5:** Implant volume and surface.

	Only Anodizing	Only Etching	Sandblasting by Al_2_O_3_ + Etching	Sandblasting by TiO_2_ + Etching
implant volume [mm^3^]	9.45	8.91	9.46	9.54
implant surface [mm^2^]	60.82	69.11	66.85	63.29

## Data Availability

The data are unavailable due to privacy or ethical restrictions.
